# Fiber-Optic Multipoint Sensor System with Low Drift for the Long-Term Monitoring of High-Temperature Distributions in Chemical Reactors

**DOI:** 10.3390/s19245476

**Published:** 2019-12-12

**Authors:** Franz J. Dutz, Andreas Heinrich, Rolf Bank, Alexander W. Koch, Johannes Roths

**Affiliations:** 1 Photonics Laboratory, Munich University of Applied Sciences, Lothstrasse 34, 80335 Munich, Germany; 2 MAN Energy Solutions SE, Werftstrasse 17, 94469 Deggendorf, Germany; andreas.heinrich@man-es.com (A.H.); rolf.bank@man-es.com (R.B.); 3 Institute for Measurement Science and Sensor Technology, Technical University of Munich, Theresienstrasse 90, 80333 Munich, Germany; a.w.koch@tum.de

**Keywords:** fiber Bragg grating, multipoint measurement, temperature profile monitoring, regenerated grating, high-temperature sensing, chemical reactors

## Abstract

A low-drift fiber-optic sensor system, consisting of 24 regenerated fiber Bragg gratings (RFBG), equally distributed over a length of 2.3 m, is presented here. The sensor system can monitor spatially extended temperature profiles with a time resolution of 1 Hz at temperatures of up to 500 °C. The system is intended to be used in chemical reactors for both the control of the production ramp-up, where a fast time response is needed, as well as for production surveillance, where low sensor drifts over several years are required. The fiber-optic sensor system was installed in a pilot test reactor and was exposed to a constant temperature profile, with temperatures in the range of 150–500 °C for more than two years. During this period, the temperature profile was measured every three to five months and the fiber-optic temperature data were compared with data from a three-point thermocouple array and a calibrated single-point thermocouple. A very good agreement between all temperature measurements was found. The drift rates of the 24 RFBG sensor elements were determined by comparing the Bragg wavelengths at a precisely defined reference temperature near room temperature before and after the two-year deployment. They were found to be in the range of 0.0 K/a to 2.3 K/a, with an average value of 1.0 K/a. These low drift rates were achieved by a dedicated temperature treatment of the RFBGs during fabrication. Here, the demonstrated robustness, accuracy, and low drift characteristics show the potential of fiber-optic sensors for future industrial applications.

## 1. Introduction

By leaving the laboratory-scale, multipoint fiber-optic temperature sensing based on fiber Bragg gratings has gained significance for industrial applications. The main advantages arise from their immunity to electromagnetic interference, small size, and wavelength multiplexing capability. The latter is of special interest, because it enables a large number of measuring points in a single sensor cable, due to the wavelength-encoded sensor signal. When compared to conventional electrical sensors, such as thermocouples, the technology of multipoint FBG temperature sensing dramatically reduces the cabling efforts.

In many fields of industrial applications, such as chemical reactors or gas turbines, the maximum temperatures exceed 400 °C. This is out of the durability range of standard type I FBGs, because those gratings strongly decay at high temperatures [1,2]. Amongst others, type II FBGs inscribed with femtosecond (fs) lasers [3,4,5] and regenerated FBGs (RFBGs) [6,7,8] have been reported to be suitable for temperatures up to 1200 °C. Most often, type II FBGs are inscribed with high-intensity femtosecond laser beams by phase masks (type II-PM) [3,9] or by a point-by-point (type II-PbP) [10] technique, and their main features are a high grating reflectivity, inherent temperature stability, and high initial tensile strength. The disadvantages of type II FBGs arise from their polarization sensitivity [10,11] and strong cladding mode coupling [5,10], which can limit their multiplexing capabilities. Type II-PbP gratings show significant wavelength drift when exposed to high temperatures [12,13]. For type II-PM FBGs, a stabilization of the wavelength drift after an annealing procedure of 100 h at 1000 °C has been reported [14]. Due to temperature treatments, a thermal-induced glass corrosion and thus a reduction of the tensile strength has to be taken into account.

Regenerated fiber Bragg gratings (RFBGs) emerge from type I gratings in H_2_-loaded fibers after an annealing process at temperatures in the range of 800 °C to 1100 °C [7,15]. Their uniform spectral line shapes and the lack of cladding mode coupling make RFBGs perfectly suited for multiplexing applications. When compared to type II FBGs, RFBGs typically exhibit lower values of grating reflectivity. However, the high-temperature annealing process that is necessary to form RFBGs leads to a reduction of their tensile strength, but it has the advantageous consequence that this type of high-temperature resistant grating shows low drift rates of the Bragg wavelengths when compared to type II-PbP FBGs [12,13]. The issue of the mechanical robustness of the fiber-optic sensor system has to be addressed with appropriate sensor packaging and with specific measures during the annealing process. For the application reported in this article, the priority lies in the low drift characteristics and the multiplexing capabilities, and this is why we use regenerated gratings here. 

So far, industrial employments of regenerated and type II grating-based multipoint sensing have been reported for gas turbines [12,16,17], various combustor systems [5], nuclear reactors [13] and a test facility of the chemical industry [12]. In the chemical industry, catalytic fixed-bed reactors are widely used for the large-scale synthesis of basic chemicals and intermediates such as methanol, phthalic anhydride [18], and maleic anhydride [19]. Another emerging field of application lies in the production of synthesis gas and substitute natural gas (methanation) for carbon dioxide reduction purposes [20]. Common arrangements of catalytic fixed-bed reactors are tubular reactors, where the chemical conversion takes place in the form of heterogeneously catalyzed gas-phase reactions in 3 m to 10 m long, vertically oriented reaction tubes, which are filled with catalyst particles and a heat transfer medium (e.g., molten salt) which externally circulates the reaction tubes [21]. Generally, the quantity of the tubes varies in dependence on the purpose, such as from one tube in pilot-scale plants, where the optimal process parameters concerning output, efficiency and product quality are determined, to several ten thousand tubes in the subsequent tube bundle reactors for large-scale production [21].

With strongly exothermic successive reactions, such as partial oxidations, reaction kinetics lead to the development of a characteristic axial temperature profile with a pronounced temperature maximum (a so-called hot spot), typically located close to the inlet of the reactor [21]. Especially during the start-up process, there is a risk of a runaway reaction, which means that the reaction changes from, for example, conversion to the desired product to total combustion of the reactants, and thus the hot spot temperature rapidly increases to values far beyond 700 °C [21]. In addition, the reaction front may move to the reactor entrance within a few seconds and can cause detonation, resulting in serious damage to the catalyst and the facility [21]. Therefore, in reactors bearing temperature-sensitive and critical processes, there is a need for fast measurements of the axial temperature distribution over the complete vertical extension of the reactors, with a high spatial resolution for localizing and preventing critical process conditions. This enables an optimal start-up process, but, on the other hand, the long-term stability is also of utmost importance. A fixed-bed reactor operates continuously without any shut-down during the lifetime of the catalysts, which is typically several years. During this period, the sensor system should maintain uncertainties of a few degrees and no recalibration or replacement of the sensor system should be necessary.

To gain access for temperature measurement, selected reactor tubes are axially equipped with protective tubes that demarcate the temperature probe from the reaction volume. The outer diameters of the protective tubes have to be as small as possible to ensure representative conditions when compared to unequipped reactor tubes. Conventionally, the temperature measurements are performed with single- or multi-point thermocouples that are inserted in the protective tubes, and the available cross section strongly limits the number of thermocouples. An increase in the spatial resolution is possible by axially moving the thermocouples via an automatic positioning device [22]. This enlarges the acquisition time for the complete profile to several minutes and it means that there is a systematic time offset of a few minutes within the measured temperatures of a profile. Besides, the mechanism is mechanically sensitive, and the space required is usually too much to be applicable in industrial facilities. In contrast, fiber-optic multipoint temperature sensing allows measuring the axial temperature distribution with both high spatial and high temporal resolution simultaneously, combined with miniaturized sensor design and high accuracy [12]. This technology is particularly attractive for industrial process control [23].

In this article, we demonstrate the suitability of multipoint RFBG sensing for applications in catalytic fixed-bed tubular reactors by measuring high-temperature profiles over a length of 2.3 m in a representative industrial environment for more than two years. The issues addressed here are the design of the sensor packaging, the measurement accuracy, and the validation of the RFBG-based temperature data with thermocouples.

Moreover, the verification of the long-term stability, regarding mechanical robustness and temperature drift of the fiber-optic sensor system is of special interest, because these characteristics are generally important for industrial applications. For the measurements, we used a 4-m-long pilot reactor from MAN Energy Solutions SE in Deggendorf, Germany. Here, three electrical heaters created a typical temperature distribution along two parallel protective tubes, with temperatures of up to 500 °C. In the first protective tube, there was a conventional temperature measurement system based on moveable thermocouples for reference data. In the second protective tube, a RFBG multipoint temperature sensor system consisting of 24 elements evenly distributed over 2.3 m was installed. For more than two years, temperatures were kept constant and repeated measurements of the axial temperature profile were carried out. A description of the production process of the RFBG sensor arrays, including the regeneration procedure and the sensor calibration, is given below. Subsequently, the test setup is shown in detail and the results of the long-term measurements are presented. Concluding, the results are discussed in matters of temperature accuracy and temperature drift.

## 2. Production of RFBG Sensor Arrays

### 2.1. Fabrication of Seed Grating Sensor Arrays

**Figure 1:**
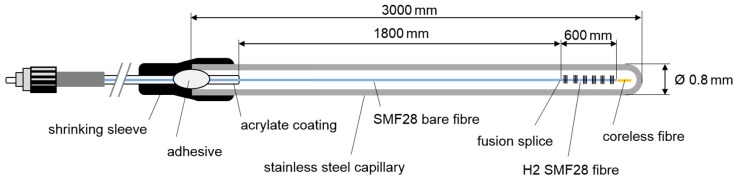
Schematic of the sensor packaging of a six-element regenerated fiber Bragg grating (RFBG) sensor array. Four identical arrays were produced.

**Figure 2:**
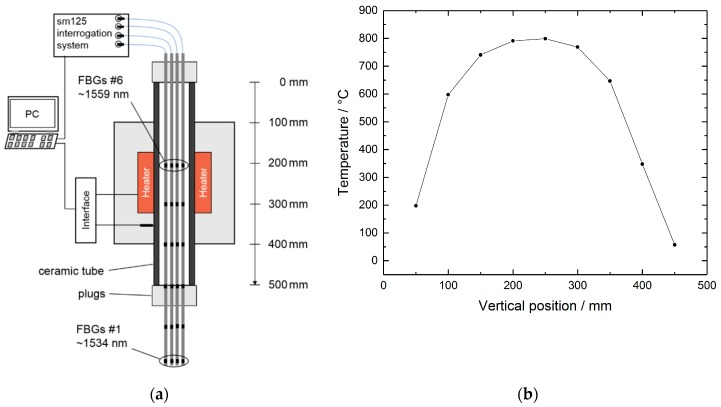
(**a**) Schematic of the high-temperature tube furnace used for regeneration of the RFBG sensors. (**b**) Vertical temperature distribution inside the tube furnace at a set point of 800 °C, measured with a RFBG temperature sensor. The total length of the ceramic tube is 500 mm, and 0 mm corresponds to the top of the tube.

**Figure 3:**
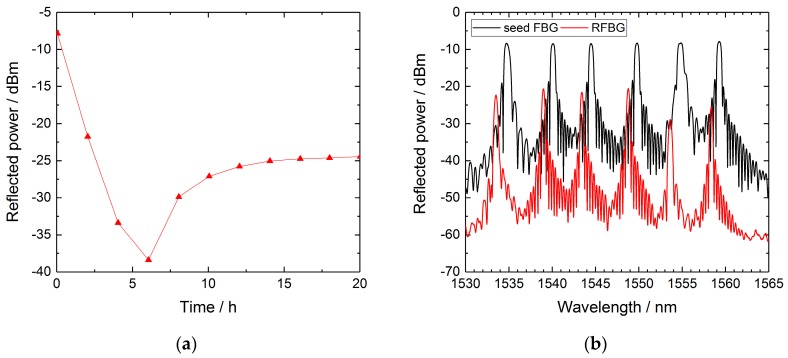
(**a**) Time series of the reflected Bragg peak power of FBG #6 (1558 nm) of array A1. (**b**) Spectra of sensor array A1 at room temperature before and after the regeneration procedure.

Figure 1 shows a schematic of the FBG sensor array. Over a length of 600 mm, the acrylate coating of an H_2_-loaded (180 bar, 2 weeks, 25 °C), standard single-mode fiber SMF28 was removed, and six seed FBGs were inscribed with a KrF Excimer laser (MLI-200, MLase AG, Germering, Germany) using the phase mask method. The FBGs had lengths of 2.6 mm and the positions of the FBGs were equally separated by 100 mm. The Bragg wavelengths of the seed gratings ranged from 1534 nm to 1559 nm (FBG #1 to #6, see Figure 2a and Figure 3b) and the spectral separations were about 5 nm, in order to avoid spectral overlapping, even if large temperature gradients between the FBG locations were present. A short piece of coreless fiber was spliced to the end of the sensor fiber to avoid disturbing interference effects caused by back-reflected light from the rear end of the fiber. Then, the sensor fiber was spliced to a standard SMF 28 fiber with an 1800-mm-long bare fiber segment. The acrylate coating was removed from the designated fiber sections, because it would burn at the temperatures expected there. 

In order to protect the fiber from mechanical degradation and to avoid that external strain is transferred to the fiber, the fiber sensor array was loosely installed in a stainless-steel capillary, with an outside diameter of 0.8 mm and about three meters in length. Glue sealed the proximal end of the capillary and fixed the fiber to the capillary at this point. Additionally, a piece of shrinking sleeve protected the fiber from folding and breaking at the entrance of the capillary. The distal end of the steel capillary was closed by laser beam welding. Four identical FBG sensor arrays were produced (array identifiers A1, A2, A3, and A4) for the measurements described. 

### 2.2. Regeneration Procedure

Generally, the fabrication process of RFBGs consists of thermal treatment of the seed gratings for several hours at temperatures in the range of about 800 °C to 1100 °C. This process can be performed either with linearly or gradually increasing temperatures [6,7,24] or under isothermal conditions [12,15,25]. For regeneration of the seed grating sensor arrays, an isothermal scheme at 800 °C for several hours was used here. Therefore, the four packaged sensor arrays were suspended in a vertically oriented, high-temperature tube furnace ROS 20-250-12 (ThermConcept GmbH, Bremen, Germany), as shown in Figure 2a. The furnace consisted of a central ceramic tube of 500 mm in length, with a 20 mm inner diameter, with electrical heaters and isolation. Ceramic plugs at the upper and lower ends of the tube avoided temperature deviations due to air fluctuations, and the four arrays were threaded through small notches in the plugs. In this configuration, and at a set point of 800 °C, a vertical temperature distribution as shown in Figure 2b was established inside the oven. In a first step, the upper two FBG elements of each array were located at vertical positions of 200 mm and 300 mm, with local temperatures of 790 °C and 765 °C, respectively (see Figure 2b). After regeneration of the upper two FBG elements, the arrays were lifted and the two FBGs in the middle of the arrays were positioned at 200 mm and 300 mm for regeneration, respectively. This step was also repeated for the remaining two FBG elements of the arrays, so that all the single FBGs experienced thermal annealing at 765 °C to 790 °C for at least twenty hours (see Figure 3a).

During the regeneration procedure, a four-channel sm125 interrogation system (Micron Optics, Atlanta, USA) measured the reflection spectra of the RFBG sensor arrays. Figure 3a exemplarily shows the time series of the reflected Bragg peak power of FBG #6 (1559 nm) of array A1 during annealing. In the beginning, the reflectivity of the grating showed typical thermal decay. After about 6 h, the grating started to regenerate, and after 20 h, the reflectivity of the RFBG approached a maximum, indicating a completed regeneration process. Figure 3b depicts the spectra of sensor array A1 at room temperature (approximately 24 °C) before and after regeneration. As can be seen, the Bragg wavelengths exhibited a blue shift of about 1.1 nm due to the regeneration procedure and the regeneration efficiencies, expressed by the ratios of peak reflected power to pristine peak reflected power, ranged from 1% to 6%. This is lower than the typical values reported for regeneration experiments with seed grating arrays, where the gratings regenerated to about 10% of their pristine peak power [26,27]. Despite the low peak reflectivity values of the RFBGs, the signal-to-noise ratio (SNR) was still larger than 25 dB, enabling precise detection of the Bragg peaks (Figure 3b).

### 2.3. Wavelength vs. Temperature Calibration

For the large RFBG sensor arrays used here, no calibration device with a sufficiently large heat zone was available to directly calibrate the produced RFBG sensor arrays. Lindner et al. [26] showed that different RFBGs, which were fabricated with the same type of optical fiber with the same grating period (the same Bragg wavelength of ~1548 nm at reference temperature), which experienced the same annealing procedure, exhibited equal dependencies of their Bragg wavelength shifts on temperature. They reported a generalized non-linear calibration function for temperatures ranging from about 20 °C to 800 °C, with an uncertainty of ±1.5 K [26], which is expressed as follows:

*λ_B_*(*T*) = *λ_B_*(0) + *AT* + *BT*^2^ + *CT*^3^ + *DT*^4^ + *ET*^5^, (1)

where *λ_B_*(*T*) is the Bragg wavelength of a given RFBG at temperature *T* in °C, *λ_B_*(0) is an individual parameter, which represents the Bragg wavelength of the respective RFBG at zero centigrade, and *A* to *E* are general parameters whose values are listed in Table 1 [26].

If the individual Bragg wavelength of a RFBG element *λ_B_*(*T_R_*) at reference temperature *T_R_* is known precisely, the parameter *λ_B_*(0) can be calculated according to the following equation:

*λ_B_*(0) = *λ_B_*(*T_R_*) − *AT_R_* − *BT_R_*^2^ − *CT_R_*^3^ − *DT_R_*^4^ − *ET_R_*^5^. (2)

For the RFBG sensor arrays used in the research described here, the same type of optical fiber and fabrication process was used as in [26]. Only the Bragg wavelengths of the RFBG arrays were in the range of 1533 nm to 1558 nm (see Table 2). Despite the differences in Bragg wavelengths, the generalized calibration function was applied here, too. For determining *λ_B_*(*T_R_*), the arrays were placed in the tube furnace while the furnace was switched off, in order to minimize temperature fluctuations due to air drafts in the laboratory. The actual temperature inside the tube was measured with a calibrated Pt100 thermistor (JUMO GmbH and Co. KG, Fulda, Germany) with an uncertainty of ±0.1 K, and was found to be 23.9 °C. The Bragg wavelengths were measured with a sm125 interrogation system (Micron Optics, Atlanta, USA), which allowed determination of the Bragg wavelengths with an uncertainty of ±2 pm. The Bragg wavelengths of the RFBGs at a reference temperature are given in Table 2 in Section 4. As mentioned, the generalized calibration function is valid for RFBGs comprising a Bragg wavelength of 1548 nm. Compared to [26], the RFBG wavelengths used here differed by up to −15 nm at the reference temperature. In this case, a dependency of the RFBG temperature sensitivity on the wavelength due to thermal expansion of the fiber and the grating period has to be considered. Assuming a linear relationship, temperature deviations *δT* arise from the relative Bragg wavelength difference according to the following equation:

*δT/T* = ±15 nm/1548 nm = ±9 × 10^−3^. (3)

Thus, we expect an expanded uncertainty of ±4.5 K at temperatures of up to 500 °C when applying the generalized calibration function, expressed in Equation (1), with Bragg wavelengths in the range from 1533 nm to 1558 nm.

## 3. Long-Term Deployment in a Tubular Pilot Reactor

**Figure 4:**
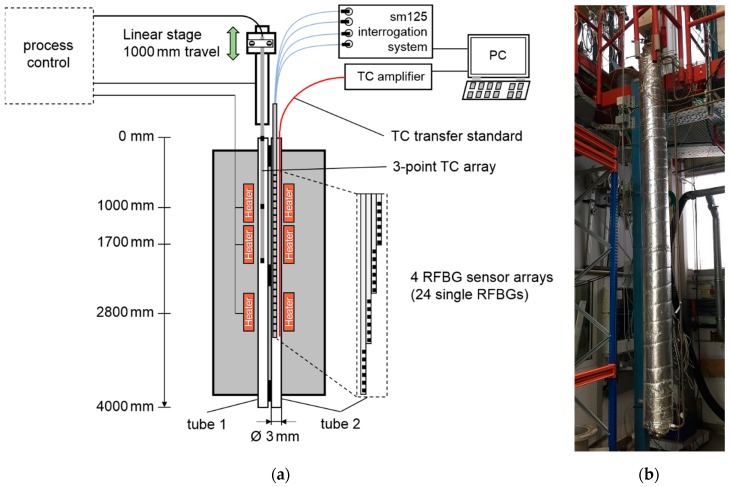
(**a**) Schematic of the measurement setup. The pilot test reactor consisted of two closely spaced protective tubes, three electrical heaters and isolation. In tube 1, a three-point type K thermocouple (TC) array was installed. The linear stage was used to shift the TC array vertically. In tube 2, the RFBG sensor system was installed. For the last measurement, a calibrated single-point thermocouple (TC transfer standard) was additionally inserted and translated in tube 2. (**b**) Photograph of the pilot reactor.

For demonstrating the suitability of RFBG fiber sensors for long-term temperature profile sensing in tubular reactors, we deployed the RFBG sensor arrays in a pilot test reactor as shown in Figure 4. This setup enabled the generation of temperature profiles, which were typical for catalytic gas-phase reactions regarding the temperature range and the spatial extension. Electrical heaters at three different vertical positions (~1000 mm, ~1700 mm, ~2800 mm) created a vertical temperature profile along two parallel, closely-spaced protective tubes, each of about 4000 mm in length and 3 mm in inner diameter (Figure 4a). The tubes were welded on each other at spots on the top, middle, and bottom of the reactor. In the first protective tube (tube 1), an array of three type K thermocouples (TC) of a 2 mm outer diameter and tolerance class 1 (Rössel-Messtechnik GmbH, Werne, Germany) was installed. The TC sensor array was mounted on a linear stage capable of one-meter travel (Figure 4a) and the elements were separated by one meter. With this translation mechanism, it was possible to measure the vertical temperature profiles over a total range of 3000 mm with a high spatial resolution by shifting the thermocouples vertically inside the protective tube [22]. The vertical step size was set to 20 mm and the dwell time at each position was two minutes. This allowed the system to achieve thermal equilibrium before the temperature measurement was performed with the thermocouples. The time needed for acquiring a complete temperature profile was 100 minutes. 

In the second protective tube (tube 2), the four RFBG sensor arrays were installed with vertical offsets to each other, so the 24 RFBG elements covered a length of 2300 mm with spacings between adjacent measurement points of about 100 mm (see the inset of Figure 4a). The diameter of the four stacked RFBG arrays was less than 2 mm. It is worth noting that the length of the fiber-optic sensor can be easily extended by adding additional RFBG arrays. A four-channel sm125 interrogation system (Micron Optics, Atlanta, USA) provided the RFBG spectral data. The temperature values were automatically calculated from the measured Bragg wavelengths by numerically inverting the fifth-order polynomial calibration function, expressed in Equation (1). The time needed to acquire a complete temperature profile with the RFBG arrays was about one second.

**Figure 5:**
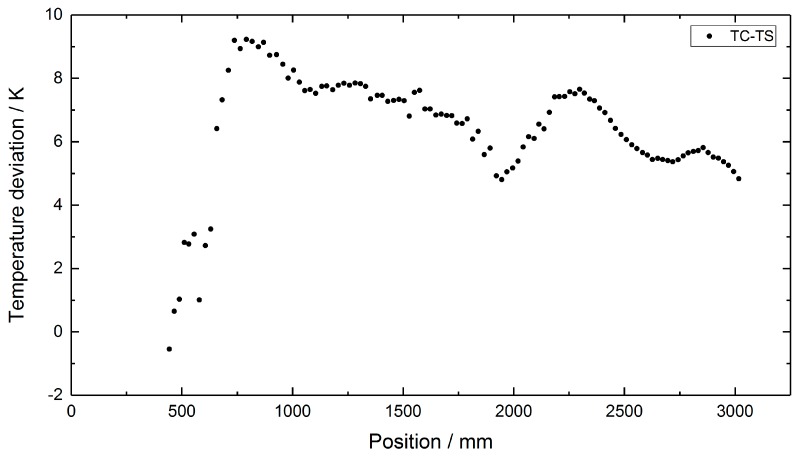
Position-dependent temperature deviations between the thermocouples in tube 1 and the calibrated TC transfer standard in tube 2, as measured on 24 April 2019.

**Figure 6:**
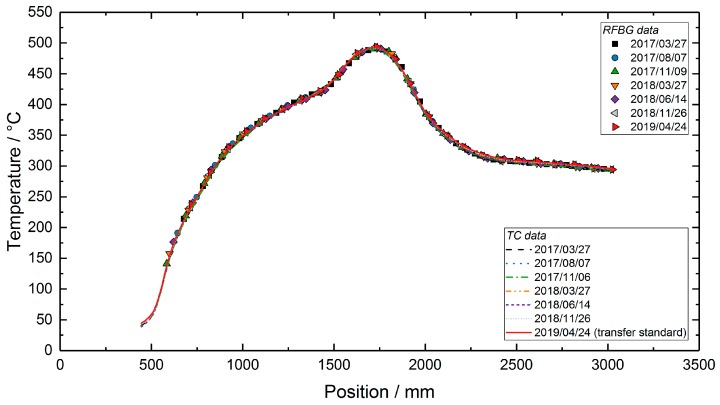
Temperature profiles in the test facility, measured during an operating period of 758 days. The TC data were corrected by the values shown in Figure 5.

During the long-term deployment, the three heaters were set to constant temperature values of 350 °C, 500 °C and 300 °C, respectively. From March 2017 to April 2019, the test facility was kept at this constant temperature profile and the RFBG sensor arrays remained inside the facility at high temperatures. Seven individual measurements of the temperature profile were performed during the two-year period at intervals of three to five months. During the last measurement on 24 April 2019, an additional calibrated thermocouple with an outer diameter of 0.5 mm (TC Mess-und Regeltechnik GmbH, Mönchengladbach, Germany) was used as a transfer standard. It had a temperature uncertainty of ±2.0 K at 500 °C and was inserted in tube 2 (Figure 4a) beside the RFBG arrays. This thermocouple was manually shifted in steps of 20 mm to obtain the axial temperature distribution with high accuracy. This measurement was done to identify systematic uncertainties, such as possible temperature gradients between both protective tubes. By subtracting the TC transfer standard data (tube 2) from the TC array data (tube 1) of the same day (24 April 2019), position-dependent temperature deviations of up to 10 °C between both tubes were obtained (Figure 5). Then, these values were used to correct the TC-based temperatures measured in tube 1 at the other days. During the operating period of more than two years (758 days), several workings on the pilot plant were carried out. As a result, the external parts of the RFBG sensor arrays were subjected to strong agitations. This caused vertical shifts of the RFBG sensor arrays, and their positions relative to the protective tube changed by up to 100 mm between successive measurements. For each measurement, the locations of the RFBG arrays were determined and the appropriate position of each RFBG sensor element was updated. Figure 6 depicts the results of the long-term deployment. It shows all temperature profiles measured by the RFBG sensor system and the corrected data of the moveable three-point TC array at the respective dates. The RFBG-based temperature data are in good accordance with the corrected TC-based data. After two years of operation, we removed the RFBG sensor system from the pilot reactor and brought it back to the laboratory for investigating possible wavelength drifts (see Section 4).

## 4. Discussion

**Figure 7:**
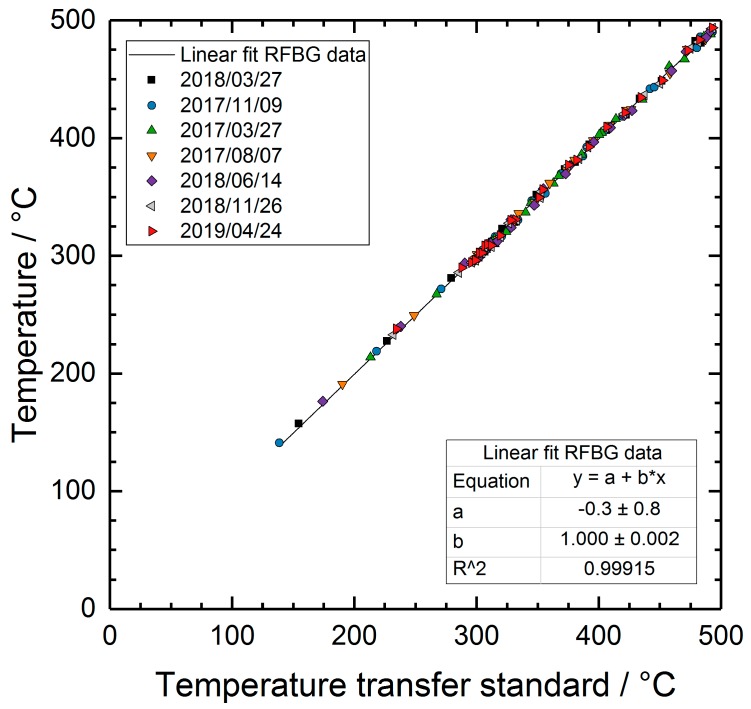
Temperature values measured by the RFBG sensor system versus reference temperatures measured by the transfer standard. The solid line shows a fit of a linear function to the data.

In order to check the employed calibration function, all the RFBG-based temperatures measured during the two-year deployment were related to the transfer standard temperatures obtained on 24 April 2019. Figure 7 shows the resulting plot with a linear function fitted to the data. The slope and the offset of the linear fit equates with (1.000 ± 0.002) K and (−0.3 ± 0.8) K, respectively. This demonstrates that the calibration function was valid during the long-term application.

**Figure 8:**
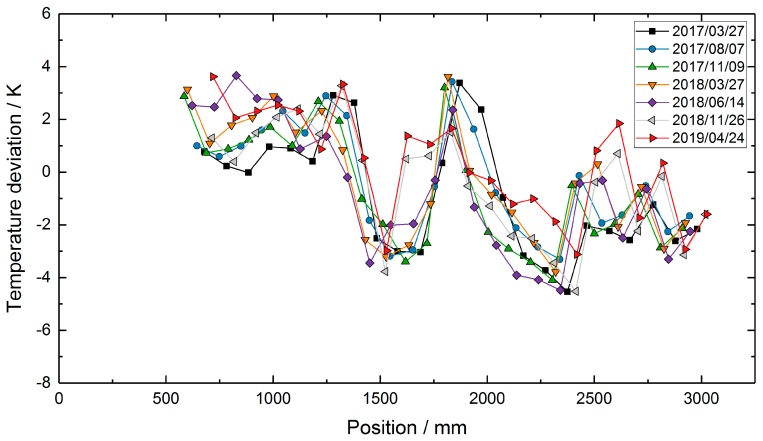
Temperature deviations between the RFBG-based temperature profiles and the measurement with the TC transfer standard on 24 April 2019.

To assess the sensor performance in terms of temperature accuracy, the temperature profile measured with the TC transfer standard on 24 April 2019, was subtracted from the temperature profiles of the RFBG sensor system (see Figure 8). If there was no measuring point at the same position, the corresponding temperature of the single-point TC transfer standard was calculated by linear interpolation between two neighboring values. Figure 8 depicts the temperature deviations between the transfer standard and the RFBG data. Here, the differences were in the range of ±4 K. The deviations of ±4 K in the RFBG data are within the uncertainty of the RFBG calibration procedure (±4.5 K, see Section 2.3). In addition to that, a drift of the RFBG sensors or instabilities of the setup might also contribute to the observed differences.

**Figure 9:**
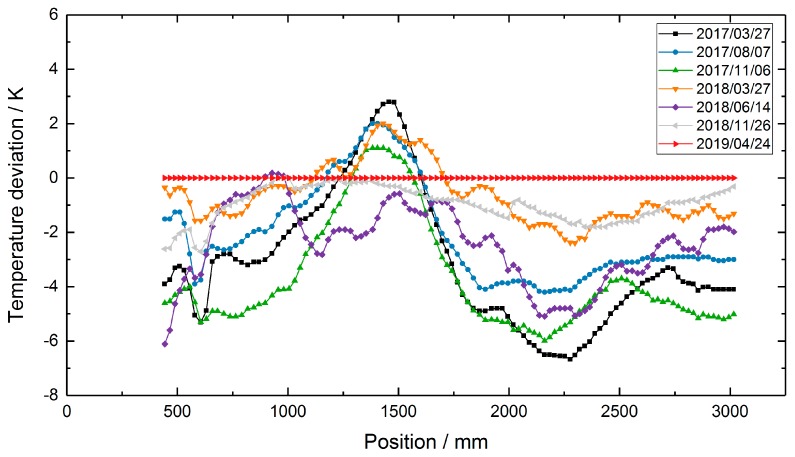
Temperature deviations between the corrected TC-based temperature profiles and the measurement with the TC transfer standard on 24 April 2019.

In order to further characterize the setup used and the three-point TC array, the three-point TC array data were corrected for the temperature differences between tube 1 and tube 2. Figure 9 shows the differences of all corrected three-point TC data taken during the two-year campaign and the single-point TC data from 24 April 2019. These differences are in the range of +3 K to −7 K and thus slightly larger than those of the RFBG-based data.

**Figure 10:**
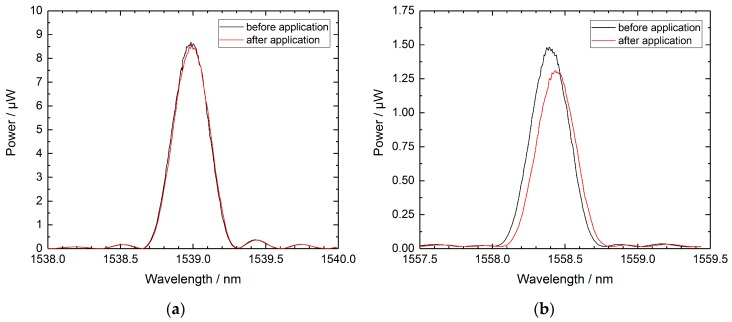
RFBG spectra at reference temperature of about 24 °C before and after two years of operation at temperatures up to 500 °C. (**a**) RFBG #2 of array A1 shows no changes. (**b**) RFBG #6 of array A2 comprises a red shift of 48 pm and a slight degradation in reflected power.

**Figure 11:**
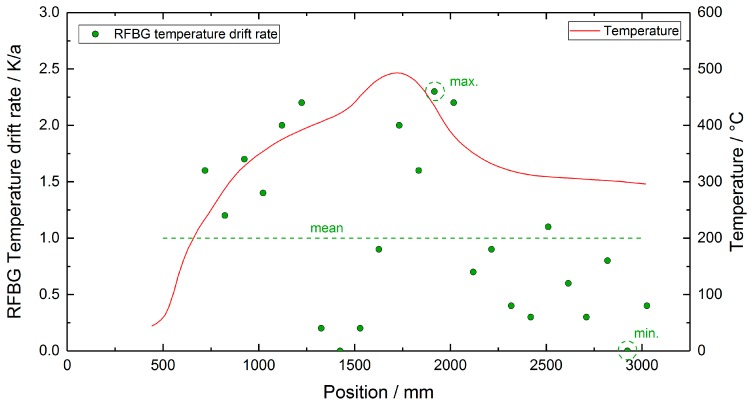
Temperature drift rates of the RFBG elements and long-term temperature profile. The drift rates spread from 0.0 K/a (min.) to 2.3 K/a (max.) and the dashed line corresponds to the average drift rate of 1.0 K/a.

Due to the uncertainties in the calibration procedure, the data shown in Figure 7 and Figure 8 give no clear evidence of a temperature drift in the RFBG sensors. In order to investigate a possible sensor drift of the RFBGs, the RFBG arrays were removed from the test reactor, brought back to the laboratory, and the spectra of the RFBG arrays were measured at a reference temperature with the same setup and procedure that was used before the deployment (see Section 2.3). In Table 2 there is a summary of the Bragg wavelengths at reference temperature measured before and after the installation in the test reactor. It can be seen that all RFBG elements still functioned when back in the laboratory and wavelength shifts between 0 pm and 48 pm occurred during the two years at high temperatures. Figure 10 exemplarily shows the spectra of the best and worst cases found: RFBG #2 of array A1 seems to be completely unaltered (see Figure 10a), whereas RFBG #6 of array A2 comprises a slight degradation of its reflectivity and it showed a wavelength shift of 48 pm (see Figure 10b). When considering a sensitivity of 10 pm/K at reference temperature, this corresponds to a temperature drift of 0 K to 4.8 K during 758 days of operation at high temperatures. Table 2 also lists the temperature drift per year (365 days), as calculated by linear interpolation. In order to check, if the drift depends on the temperature employed, Figure 11 depicts the drift rates of the RFBGs together with the long-term temperature profile. In Figure 11, no distinct dependency of the drift rate on the long-term operating temperature can be found. The drift rates range from 0.0 K/a to 2.3 K/a, and the average amounts to 1.0 K/a. 

So far, there are only a few publications on the drifts of RFBGs at high temperatures that are based on time scales of one year or more. In [13], Laffont et al. reported on the long-term annealing of four packaged RFBG sensors at temperatures ranging from 760 °C to 890 °C. After 9000 h of isothermal annealing, the RFBG elements experienced wavelength shifts larger than 500 pm, corresponding to temperature drift rates of more than 30 K/a [13]. This is considerably higher than the maximum drift rate of 2.3 K/a found in the present study, but this may be attributed to the significantly higher operating temperatures in [13].

## 5. Conclusions

A fiber-optic multipoint sensor system based on 24 RFBG sensor elements that were equally distributed over a length of 2.3 m has been presented and characterized during a long-term test procedure. The RFBG sensor system consisted of four arrays with six sensor elements each, and the arrays were packaged in three-meter-long stainless-steel capillaries of a 0.8 mm outer diameter. The RFBG sensors were exposed to a constant temperature profile that ranged from 150 °C to 500 °C for a period of 758 days. Temperature profile measurements at a 1 Hz temporal resolution were performed every three to five months. They revealed a good agreement with the simultaneously conducted thermocouple-based measurements. This verified the calibration methodology used, which consisted of determining a reference wavelength at a reference temperature for each sensor element and applying the same generalized calibration function to all sensor elements. The generalized calibration function described the Bragg wavelength changes with temperature and was experimentally determined with other single-point RFBG sensor elements of the same type and of the same fabrication procedures. The temperature-induced drift of the RFBG elements was investigated by comparing the Bragg wavelengths measured at a precisely determined reference temperature before and after the long-term high-temperature deployment. The drift rates found ranged from 0.0 K/a to 2.3 K/a. The RFBG sensor arrays kept up for more than two years, indicating sufficient mechanical robustness of the fiber sensors and their packaging. Overall, the successfully performed measurements demonstrate the capability and reliability of RFBG sensor arrays for applications in chemical reactors and similar industrial applications.

## 6. Patents

The use of fiber-optic temperature sensors for monitoring heterogeneously catalyzed gas-phase reactions in tubular reactors is registered as an international patent with the German Patent and Trademark Office (Deutsches Patent- und Markenamt, DPMA): WO2016097190A1 (FIBRE-OPTIC TEMPERATURE MEASUREMENT IN A CATALYST MATERIAL), 17 December 2015.

## Figures and Tables

**Table 1:** Parameters of the generalized calibration function shown in Equation (1) [26].

Parameter	*A*/nm·°C^−1^	*B*/nm·°C^−2^	*C*/nm·°C^−3^	*D*/nm·°C^−4^	*E*/nm·°C^−5^
Value	8.81 × 10^−3^	1.45 × 10^−5^	−2.01 × 10^−8^	1.9 × 10^−11^	−7.5 × 10^−15^

**Table 2:** Bragg wavelengths of the RFBG sensor elements measured at reference temperature *T_R_* of about ~24 °C before (*λ_B, start_*) and after (*λ_B, end_*) 758 days at temperatures between 150 °C and 500 °C. The shifts of the Bragg wavelengths were obtained by subtracting the start values from the end values. From the wavelength shifts, the temperature drift rates were calculated by considering a sensitivity of 10 pm/K (sensitivity at room temperature, according to Equation (1)).

Array ID	RFBG Number	*λ_B, start_* (*T_R_*)/nm	*λ_B, end_* (*T_R_*)/nm	Δ*λ_B_*/pm	Temperature Drift Per Year/K·a^−1^
A1	#1	1533.512	1533.521	9	0.4
A1	#2	1538.990	1538.990	0	0.0
A1	#3	1543.428	1543.444	16	0.8
A1	#4	1548.778	1548.785	7	0.3
A1	#5	1553.626	1553.639	13	0.6
A1	#6	1558.352	1558.375	23	1.1
A2	#1	1533.524	1533.531	7	0.3
A2	#2	1538.954	1538.962	8	0.4
A2	#3	1543.460	1543.479	19	0.9
A2	#4	1548.758	1548.773	15	0.7
A2	#5	1553.794	1553.840	46	2.2
A2	#6	1558.391	1558.439	48	2.3
A3	#1	1533.542	1533.575	33	1.6
A3	#2	1538.956	1538.997	41	2.0
A3	#3	1543.479	1543.498	19	0.9
A3	#4	1548.816	1548.820	4	0.2
A3	#5	1553.747	1553.748	1	0.0
A3	#6	1558.382	1558.386	4	0.2
A4	#1	1533.493	1533.538	45	2.2
A4	#2	1538.970	1539.011	41	2.0
A4	#3	1543.495	1543.525	30	1.4
A4	#4	1548.813	1548.848	35	1.7
A4	#5	1553.846	1553.870	24	1.2
A4	#6	1558.509	1558.543	34	1.6
